# Bis(2,6-dimethyl­pyridinium) tetra­bromido­zincate(II)

**DOI:** 10.1107/S1600536809015219

**Published:** 2009-04-30

**Authors:** Basem Fares Ali, Rawhi Al-Far

**Affiliations:** a Department of Chemistry, Al al-Bayt University, Mafraq 25113, Jordan; b Faculty of Information Technology and Science, Al-Balqa’a Applied University, Salt, Jordan

## Abstract

In the crystal structure of the title compound, (C_7_H_10_N)_2_[ZnBr_4_], the coordination geometry of the anion is approximately tetra­hedral and a twofold rotation axis passes through the Zn atom. The Zn—Br bond lengths range from 2.400 (2) to 2.408 (3) Å and the Br—Zn—Br angles range from 108.14 (6) to 115.15 (15)°. In the crystal structure, the [ZnBr_4_]^2−^ anion is connected to two cations through N—H⋯Br and H_2_C—H⋯Br hydrogen bonds, forming two-dimensional cation–anion–cation layers normal to the *b* axis. No significant Br⋯Br inter­actions [the shortest being 4.423 (4) Å] are observed in the structure.

## Related literature

The title salt is isotypic with the Co-analogue, see: Ali *et al.* (2008 ▶). For non-covalent inter­actions and their influence on the organization and properties of materials, see: Desiraju (1997 ▶); Desiraju & Steiner (1999 ▶); Hunter (1994 ▶); Allen *et al.* (1997 ▶); Dolling *et al.* (2001 ▶); Panunto *et al.* (1987 ▶); Robinson *et al.* (2000 ▶). For the structures of related halo-metal anion salts, see: Ali & Al-Far (2007 ▶); Al-Far & Ali (2007 ▶); Al-Far & Ali (2009 ▶). For distances and angles in [ZnBr_4_] anions, see: Gao *et al.* (2007 ▶). For cation bond distances, see: Allen *et al.* (1987 ▶).
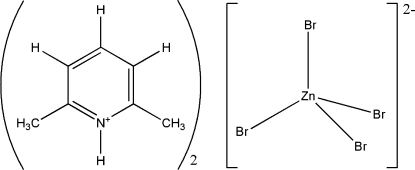


## Experimental

### 

#### Crystal data

(C_7_H_10_N)_2_[ZnBr_4_]
*M* *_r_* = 601.33
Orthorhombic, 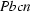

*a* = 17.237 (2) Å
*b* = 9.0754 (17) Å
*c* = 13.7302 (14) Å
*V* = 2147.9 (5) Å^3^
*Z* = 4
Mo *K*α radiation
μ = 8.58 mm^−1^
*T* = 293 K
0.30 × 0.20 × 0.20 mm

#### Data collection

Bruker P4 diffractometer
Absorption correction: numerical (*SADABS*; Bruker 2001 ▶) *T*_min_ = 0.183, *T*_max_ = 0.279
2020 measured reflections
1987 independent reflections
1850 reflections with *I* > 2σ(*I*)
*R*_int_ = 0.086
3 standard reflections every 97 reflections intensity decay: 0.01%

#### Refinement

*R*[*F* ^2^ > 2σ(*F* ^2^)] = 0.088
*wR*(*F* ^2^) = 0.177
*S* = 0.98
1980 reflections
98 parameters
H-atom parameters constrained
Δρ_max_ = 0.60 e Å^−3^
Δρ_min_ = −0.48 e Å^−3^

### 

Data collection: *XSCANS* (Siemens, 1996 ▶); cell refinement: *XSCANS*; data reduction: *SHELXTL* (Sheldrick, 2008 ▶); program(s) used to solve structure: *SHELXS97* (Sheldrick, 2008 ▶); program(s) used to refine structure: *SHELXL97* (Sheldrick, 2008 ▶); molecular graphics: *SHELXTL*; software used to prepare material for publication: *SHELXTL*.

## Supplementary Material

Crystal structure: contains datablocks I, global. DOI: 10.1107/S1600536809015219/at2771sup1.cif

Structure factors: contains datablocks I. DOI: 10.1107/S1600536809015219/at2771Isup2.hkl

Additional supplementary materials:  crystallographic information; 3D view; checkCIF report

## Figures and Tables

**Table 1:** Hydrogen-bond geometry (Å, °)

*D*—H⋯*A*	*D*—H	H⋯*A*	*D*⋯*A*	*D*—H⋯*A*
N1—H1⋯Br2	0.86	2.49	3.351 (12)	175
C7—H7*C*⋯Br1^ii^	0.96	2.91	3.861 (18)	171

Symmetry code: (ii) 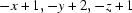.

**Fig. 1.**
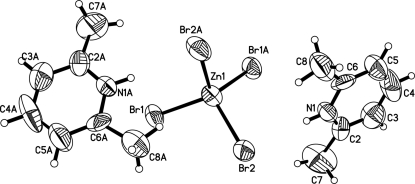
A view of the asymmetric unit of (I), with the atom-numbering scheme. Displacement ellipsoids are drawn at the 50% probability level. [Symmetry operation A: -x + 1, y, -z + 1/2].

**Fig. 2.**
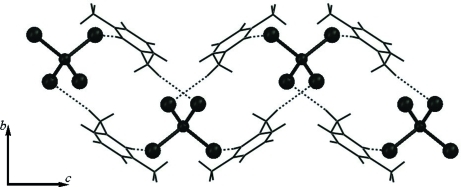
A packing diagram of (I), shows alternating stacks of anions and cations. C,N—H···Br—Zn interactions are shown as dashed lines.

## supplementary crystallographic information

### Comment

Non-covalent interactions play an important role in organizing structural units in both natural and artificial systems (Desiraju, 1997). They exercise important effects on the organization and properties of many materials in areas such as biology (Hunter 1994; Desiraju & Steiner 1999), crystal engineering (see for example: Allen *et al.*, 1997; Dolling *et al.*, 2001) and material science (Panunto *et al.*, 1987; Robinson *et al.*, 2000). The interactions governing the crystal organization are expected to affect the packing and then the specific properties of solids. In connection with ongoing studies (Al-Far & Ali, 2007; Ali & Al-Far, 2007; Ali *et al.*,  2008; Al-Far & Ali, 2009) of the structural aspects of halo-metal anion salts, we herein report the crystal structure of title compound (I).

The asymmetric unit in (I), contains half an anion and one cation (Fig. 1). The geometry of ZnBr_4_^2-^ anions is approximately tetrahedral and a twofold rotation axis passes through the Zn^II^ ion (Table 1). The Zn—Br bonds range from 2.400 (2) to 2.408 (3) Å and the Br—Zn—Br angles range from 108.14 (6) to 115.15 (15)°. The bond distances and angles fall in the range of those reported previously for compounds containing Zn—Br anions (Gao *et al.*, 2007). In the cation, the bond lengths and angles are within normal range (Allen *et al.*, 1987).

The packing of the structure (Fig. 2) can be regarded as alternating stacks of anions and stacks of cations. The anion stacks are parallel to the cation stacks, with no significant inter- and intra-stack halogen···halogen interactions [shortest Br···Br interactions being 4.4233 (35) Å]. The anions and cations are interacting significantly through extensive N—H···Br and C—H···Br hydrogen bonding involving Br^-^ anions and N—H and CH_3_ groups (Table 2). These interactions link anions and cations into two-dimensional cation···anion···cation layers normal to the crystallographic *b* axis (Fig. 2).

The N—H···Br and C—H···Br hydrogen bonding are potential building blocks for this stable supramolecular lattice. The stability of this lattice is evident in the isostructurality with the reported analogue (Ali *et al.*,  2008).

### Experimental

Warm solution of ZnCl_2_ (1.0 mmol) dissolved in absolute ethanol (10 ml) and HBr (60%, 5 ml), was mixed with a stirred hot solution of 2,6-dimethylpyridine (2 mmol) dissolved in ethanol (10 ml). The mixture was then refluxed for 2 h, and then allowed to evaporate undisturbed at room temperature. The salt crystallized over 3 d as nice colourless crystals.

### Refinement

H atoms bound to carbon and nitrogen were placed at idealized positions [C—H = 0.93 and 0.96 Å and N—H = 0.86 Å] and allowed to ride on their parent atoms with *U_iso_* fixed at 1.2 or 1.5 *U_eq_*(C,*N*).

### Crystal data

**Table:**

(C_7_H_10_N)_2_[ZnBr_4_]	*F*(000) = 1152
*M**_r_* = 601.33	*D*_x_ = 1.860 Mg m^−^^3^
Orthorhombic, *P**b**c**n*	Mo *K*α radiation, λ = 0.71073 Å
Hall symbol: -P 2n 2ab	Cell parameters from 250 reflections
*a* = 17.237 (2) Å	θ = 3.2–18.0°
*b* = 9.0754 (17) Å	µ = 8.58 mm^−^^1^
*c* = 13.7302 (14) Å	*T* = 293 K
*V* = 2147.9 (5) Å^3^	Plate, colourless
*Z* = 4	0.30 × 0.20 × 0.20 mm

### Data collection

**Table:**

Bruker P4 diffractometer	1850 reflections with *I* > 2σ(*I*)
Radiation source: fine-focus sealed tube	*R*_int_ = 0.086
graphite	θ_max_ = 25.5°, θ_min_ = 2.5°
ω scans	*h* = −1→20
Absorption correction: numerical (*SADABS*; Bruker 2001)	*k* = −1→10
*T*_min_ = 0.183, *T*_max_ = 0.279	*l* = −1→16
2020 measured reflections	3 standard reflections every 97 reflections
1987 independent reflections	intensity decay: 0.01%

### Refinement

**Table:**

Refinement on *F*^2^	Primary atom site location: structure-invariant direct methods
Least-squares matrix: full	Secondary atom site location: difference Fourier map
*R*[*F*^2^ > 2σ(*F*^2^)] = 0.088	Hydrogen site location: inferred from neighbouring sites
*wR*(*F*^2^) = 0.177	H-atom parameters constrained
*S* = 0.98	*w* = 1/[σ^2^(*F*_o_^2^) + (0.0451*P*)^2^] where *P* = (*F*_o_^2^ + 2*F*_c_^2^)/3
1980 reflections	(Δ/σ)_max_ < 0.001
98 parameters	Δρ_max_ = 0.60 e Å^−^^3^
0 restraints	Δρ_min_ = −0.48 e Å^−^^3^

### Special details

**Table:**

Geometry. All e.s.d.'s (except the e.s.d. in the dihedral angle between two l.s. planes) are estimated using the full covariance matrix. The cell e.s.d.'s are taken into account individually in the estimation of e.s.d.'s in distances, angles and torsion angles; correlations between e.s.d.'s in cell parameters are only used when they are defined by crystal symmetry. An approximate (isotropic) treatment of cell e.s.d.'s is used for estimating e.s.d.'s involving l.s. planes.
Refinement. Refinement of *F*^2^ against ALL reflections. The weighted *R*-factor *wR* and goodness of fit *S* are based on *F*^2^, conventional *R*-factors *R* are based on *F*, with *F* set to zero for negative *F*^2^. The threshold expression of *F*^2^ > σ(*F*^2^) is used only for calculating *R*-factors(gt) *etc*. and is not relevant to the choice of reflections for refinement. *R*-factors based on *F*^2^ are statistically about twice as large as those based on *F*, and *R*- factors based on ALL data will be even larger.7 reflections were rejected based on high deviation from observed ones

### Fractional atomic coordinates and isotropic or equivalent isotropic displacement parameters (Å^2^)

**Table:**

	*x*	*y*	*z*	*U*_iso_*/*U*_eq_	
Zn1	0.5000	0.7858 (3)	0.2500	0.0644 (10)	
Br1	0.60634 (9)	0.9276 (2)	0.18715 (12)	0.0652 (6)	
N1	0.3780 (7)	0.6538 (15)	0.4943 (10)	0.057 (4)	
H1	0.4205	0.6422	0.4626	0.068*	
Br2	0.54864 (10)	0.6305 (2)	0.37844 (13)	0.0812 (7)	
C2	0.3794 (11)	0.736 (2)	0.5723 (14)	0.062 (5)	
C3	0.3122 (13)	0.747 (2)	0.6241 (14)	0.087 (6)	
H3	0.3123	0.8001	0.6818	0.105*	
C4	0.2454 (14)	0.683 (3)	0.5953 (19)	0.105 (9)	
H4	0.1999	0.6943	0.6308	0.126*	
C5	0.2470 (11)	0.599 (2)	0.5103 (15)	0.093 (7)	
H5	0.2025	0.5519	0.4879	0.112*	
C6	0.3146 (12)	0.586 (2)	0.4611 (11)	0.065 (5)	
C7	0.4534 (12)	0.819 (2)	0.5998 (14)	0.134 (9)	
H7A	0.4741	0.8668	0.5431	0.200*	
H7B	0.4908	0.7507	0.6250	0.200*	
H7C	0.4416	0.8915	0.6485	0.200*	
C8	0.3264 (10)	0.498 (2)	0.3707 (13)	0.102 (7)	
H8A	0.3733	0.4416	0.3764	0.153*	
H8B	0.3302	0.5628	0.3157	0.153*	
H8C	0.2833	0.4323	0.3618	0.153*	

### Atomic displacement parameters (Å^2^)

**Table:**

	*U*^11^	*U*^22^	*U*^33^	*U*^12^	*U*^13^	*U*^23^
Zn1	0.0466 (16)	0.079 (2)	0.0675 (17)	0.000	0.0036 (16)	0.000
Br1	0.0502 (10)	0.0731 (13)	0.0722 (11)	−0.0146 (10)	0.0135 (10)	−0.0024 (12)
N1	0.041 (8)	0.073 (11)	0.057 (9)	−0.007 (8)	0.001 (8)	−0.013 (9)
Br2	0.0493 (10)	0.1111 (17)	0.0831 (12)	0.0147 (12)	0.0050 (11)	0.0377 (13)
C2	0.065 (13)	0.060 (13)	0.061 (12)	−0.004 (11)	0.009 (11)	0.010 (11)
C3	0.090 (15)	0.097 (17)	0.075 (13)	0.007 (15)	0.012 (15)	−0.012 (14)
C4	0.069 (15)	0.11 (2)	0.14 (2)	0.034 (15)	0.052 (16)	0.023 (19)
C5	0.043 (11)	0.12 (2)	0.116 (17)	0.005 (13)	0.017 (13)	0.018 (18)
C6	0.074 (13)	0.085 (15)	0.037 (10)	0.014 (13)	−0.021 (10)	0.011 (12)
C7	0.107 (18)	0.17 (2)	0.125 (18)	−0.016 (18)	−0.024 (15)	−0.080 (18)
C8	0.069 (12)	0.14 (2)	0.094 (15)	−0.031 (14)	0.008 (13)	−0.011 (16)

### Geometric parameters (Å, °)

**Table:**

Zn1—Br1	2.400 (2)	C4—C5	1.39 (3)
Zn1—Br1^i^	2.400 (2)	C4—H4	0.9300
Zn1—Br2^i^	2.408 (3)	C5—C6	1.35 (2)
Zn1—Br2	2.408 (3)	C5—H5	0.9300
N1—C2	1.31 (2)	C6—C8	1.49 (2)
N1—C6	1.333 (19)	C7—H7A	0.9600
N1—H1	0.8600	C7—H7B	0.9600
C2—C3	1.36 (2)	C7—H7C	0.9600
C2—C7	1.53 (2)	C8—H8A	0.9600
C3—C4	1.35 (3)	C8—H8B	0.9600
C3—H3	0.9300	C8—H8C	0.9600
			
Br1—Zn1—Br1^i^	115.15 (15)	C6—C5—C4	119 (2)
Br1—Zn1—Br2^i^	108.45 (6)	C6—C5—H5	120.6
Br1^i^—Zn1—Br2^i^	108.14 (6)	C4—C5—H5	120.6
Br1—Zn1—Br2	108.14 (6)	N1—C6—C5	119.8 (17)
Br1^i^—Zn1—Br2	108.45 (6)	N1—C6—C8	114.9 (17)
Br2^i^—Zn1—Br2	108.35 (16)	C5—C6—C8	125 (2)
C2—N1—C6	124.1 (15)	C2—C7—H7A	109.5
C2—N1—H1	118.0	C2—C7—H7B	109.5
C6—N1—H1	118.0	H7A—C7—H7B	109.5
N1—C2—C3	116.7 (18)	C2—C7—H7C	109.5
N1—C2—C7	120.0 (16)	H7A—C7—H7C	109.5
C3—C2—C7	123 (2)	H7B—C7—H7C	109.5
C4—C3—C2	123 (2)	C6—C8—H8A	109.5
C4—C3—H3	118.5	C6—C8—H8B	109.5
C2—C3—H3	118.5	H8A—C8—H8B	109.5
C3—C4—C5	118 (2)	C6—C8—H8C	109.5
C3—C4—H4	121.2	H8A—C8—H8C	109.5
C5—C4—H4	121.2	H8B—C8—H8C	109.5
			
C6—N1—C2—C3	3(2)	C3—C4—C5—C6	0(3)
C6—N1—C2—C7	−175.0 (17)	C2—N1—C6—C5	−2(3)
N1—C2—C3—C4	−4(3)	C2—N1—C6—C8	179.9 (15)
C7—C2—C3—C4	175 (2)	C4—C5—C6—N1	0(3)
C2—C3—C4—C5	2(3)	C4—C5—C6—C8	178.4 (19)

Symmetry codes: (i) −*x*+1, *y*, −*z*+1/2.

### Hydrogen-bond geometry (Å, °)

**Table:**

*D*—H···*A*	*D*—H	H···*A*	*D*···*A*	*D*—H···*A*
N1—H1···Br2	0.86	2.49	3.351 (12)	175
C7—H7C···Br1^ii^	0.96	2.91	3.861 (18)	171

Symmetry codes: (ii) −*x*+1, −*y*+2, −*z*+1.
